# Simultaneous RP-HPLC and HPTLC Estimation of Fluoxetine Hydrochloride and Olanzapine in Tablet Dosage Forms

**DOI:** 10.4103/0250-474X.57306

**Published:** 2009

**Authors:** Sejal Patel, N. J. Patel

**Affiliations:** S. K. Patel College of Pharmaceutical Education and Research, Department of Pharmaceutical Chemistry, Ganpat University, Kherva, Mehsana-382711, India

**Keywords:** Fluoxetine HCl, olanzapine, RP-HPLC, HPTLC, simultaneous estimation

## Abstract

A binary mixture of fluoxetine HCl and olanzapine was determined by two different methods. The first method involved determination of fluoxetine HCl and olanzapine using reversed-phase liquid chromatography using acetonitrile:methanol:0.032 M ammonium acetate buffer (45:05:50, v/v/v) as the mobile phase at a flow rate of 1.5 ml/min. Quantitation was achieved with ultraviolet detection at 235 nm over concentration ranges of 0.2-4 and 0.1-2 μg/ml; mean accuracies were 101.16±0.59 and 99.79±0.56% for fluoxetine HCL and olanzapine, respectively. The second method was based on the high performance thin layer chromatography separation of the two drugs followed by densitometric measurements of their spots at 235 nm. The separation was carried out on Merck TLC aluminium sheets of silica gel 60 F254 using acetone:methanol:triethyleamine (5:3:0.5, v/v/v), as mobile phase. The linearity was found to be in the range of 300–1000 and 150–500 ng/spot; mean accuracies were 100.95±0.52 and 99.31±0.51% for fluoxetine HCl and olanzapine, respectively. The method was successively applied to tablets because no chromatographic interferences from the tablet excipients were found. The methods retained their accuracy and precision when the standard addition technique was applied. The results obtained by applying the proposed methods were statistically analyzed.

Fluoxetine HCl (FLX) is chemically, N-Methyl-γ-[4-(trifluoromethyl) phenoxy benzenpropanamine hydrochloride[1]. Fluoxetine hydrochloride is a selective serotonin reuptake inhibitor used as an antidepressant with non-sedating properties[2]. Olanzapine (OLZ) is chemically, 2-methyl-4-(4-methyl-1-piperazinyl)-10H-thieno[2,3-b] [1,5]-benzodiazepine[1]. It is an antipsychotic agent, used in schizophrenia[2]. FLX is official in BP and USP and both describe an LC method for the estimation of fluoxetine[3,4]. A literature survey indicated spectrophotometric methods of FLX in formulations[5–7]; HPLC[8–10] and LC-MS[11] methods of FLX with norfluoxetine in plasma. Literature survey also indicated HPLC[12], HPLC-MS/ESI[13], capillary GC[14] methods for simultaneous estimation of FLX in pharmaceutical formulation with drugs like fluvoxamine, clomipramine, citalopram and paroxetine. OLZ is official in IP[15], which described an HPLC method for its estimation. Literature survey indicated spectrophotometric[16], derivative spectroscopy[17] and solid phase extraction[18] methods for estimation of OLZ. Literature survey also indicated HPLC[19,20] and LC-MS[21] methods for determination of OLZ in biological fluids. FLX and OLZ are formulated together in the form of a tablet. Literature survey revealed two methods[22,23] for simultaneous determination of these two drugs. The methods presently developed have the advantage of being more sensitive to determine both drugs concurrently by simple, accurate, rapid and precise RP-HPLC and HPTLC assays for routine analysis.

For the RP-HPLC method, the chromatography was performed on a Shimadzu (Columbia, MD) RP-HPLC instrument (LC-10AT vp) equipped with UV/Vis detector, manual injector with 20 μl volume injection loop. A Phenomenex (Torrance, CA) C18 column (250×4.6 mm id, 5 μm particle size) was used as stationary phase. For HPTLC method, a Camag system comprising of Linnomat V automatic sample applicator, Camag microlitre syringe, Camag TLC Scanner-3, Camag Win CAT software and a stationary phase precoated silica gel 60F_254_ were used. CP224S analytical balance (Sartorius) and ultra sonic cleaner (Frontline FS 4) were used. Pure samples of FLX and OLZ were kindly supplied by Intas Pharmaceuticals Ltd., Ahmedabad, India. Samples of Oleanz Fort tablets, marketed by Sun Pharmaceuticals Ltd., with each tablet containing 20 mg FLX and 10 mg OLZ were used. Triple distilled water, methanol, acetonitrile (HPLC grade), ammonium acetate, acetone, methanol, triethylamine (AR grade) were procured from S. D. Fine Chemicals, Ahmedabad, India.

For the RP-HPLC method, FLX and OLZ stock solutions (200 μg/ml and 100 μg/ml, respectively) were prepared in methanol. From these stock solutions working solutions (5 μg/ml and 2.5 μg/ml) were prepared. Accurate aliquots equivalent to 0.2-4 μg FLX from its working solution (5 μg/ml) and aliquots equivalent to 0.1-2 μg OLZ from its working solution (2.5 μg/ml) were transferred into two separate sets of 5 ml volumetric flasks and diluted to volume with methanol. Using the Shimadzu instrument, chromatograms were recorded using instrument parameters such as 1.5 ml/min flow rate at ambient temperature and the eluent monitored at 235 nm. The separation was done on a C18 column using acetonitrile:methanol:0.032 M ammonium acetate buffer (45:05:50, v/v/v) as the mobile phase. Calibration curves for both FLX and OLZ were plotted, and the corresponding regression equations were calculated.

For the HPTLC method, FLX and OLZ stock solutions (500 μg/ml and 250 μg/ml) were prepared in methanol. From these stock solutions working solutions (100 μg/ml and 50 μg/ml) were prepared. Different volumes of the working solutions (3, 4, 5, 6, 7, 8, 9 and 10 μl, equivalent to 300, 400, 500, 600, 700, 800, 900 and 1000 ng/spot of FLX and 150, 200, 250, 300, 350, 400, 450 and 500 ng/spot of OLZ) were applied, in triplicate, to the TLC plate, as bands of 5 mm width using a Camag (Switzerland) Linomat V sample applicator fitted with a Camag microlitre syringe. A constant application rate of 0.1μl/s was used. Linear ascending development of the plates to a distance of 8 cm was performed with acetone:methanol:triethyleamine (5:3:0.5, v/v/v), as the mobile phase in a twin-trough glass chamber previously saturated with mobile phase vapour for 20 min at room temperature (25°). After development the plate was scanned at 235 nm by means of a Camag TLC scanner-3 in absorbance mode, using the deuterium lamp. The slit dimensions were 5×0.45 mm and the scanning speed was 10 mm/s. After development peak area data and drug concentration data were treated by linear regression to determine linearity.

Powder from the mixed contents of 20 tablets, equivalent to 20 mg FLX and 10 mg OLZ, was transferred accurately to 50 ml volumetric flask and diluted to volume with methanol. The solution was diluted to the same concentrations of working standard solutions and treated according to the linearity for the RP-HPLC and HPTLC methods.

A simple RP-HPLC method was adopted for the simultaneous determination of FLX and OLZ either in bulk powder or in pharmaceutical formulation. The best resolution was achieved using a mobile phase consisting of acetonitrile:methanol:0.032 M ammonium acetate buffer (45:05:50, v/v/v), which gave good resolution and sensitivity of both drugs (fig. 1). A linear relation was obtained between peak area and the concentration of the two drugs in the range of 0.2-4 and 0.1-2 μg/ml for FLX and OLZ, respectively. The linear regression equations were computed as: Y= 304092X–15556, r= 0.9993 and Y= 749978X+51394, r= 0.9973, where Y is the area under the peak, X is the concentration in μg/ml, and r is the correlation coefficient. System suitability testing of the RP-HPLC method gave good relative retention time = 1.95; Theoretical plates = 6162.17 and 10002.17; Asymmetry factor (A)= 1.42 and 1.24; and tailing factor (T)= 1.32 and 1.15 for FLX and OLZ, respectively (Table 1).

**Fig. 1:**
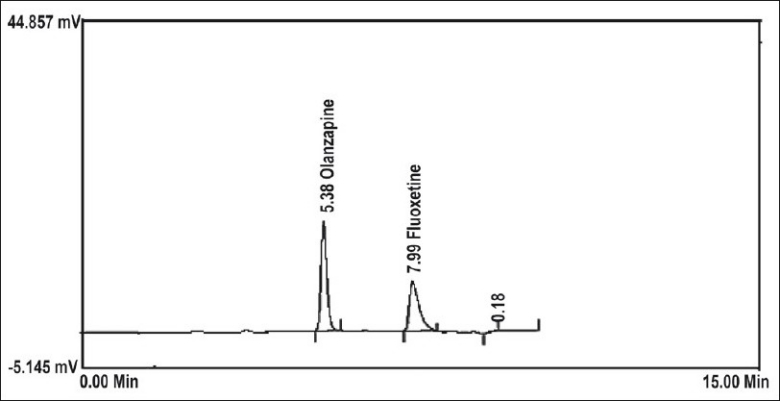
RP-HPLC chromatogram of FLX and OLZ at 235 nm. FLX is fluoxetine HCl and OLZ is olanzapine

**TABLE 1:** SYSTEM SUITABILITY TEST PARAMETERS FOR FLX AND OLZ FOR PROPOSED RP-HPLC AND HPTLC METHODS

Parameters	Proposed methods			
	
	RP-HPLC		HPTLC	
		
	FLX± % RSD^a^	OLZ± % RSD^a^	FLX± % RSD^a^	OLZ± % RSD^a^
Retention time, min	7.34±0.16	5.37±0.21	-	-
R_f_ value	-	-	0.58±0.02	0.77±0.01
Tailing factor	1.32±1.75	1.15±1.19	-	-
Asymmetry factor	1.42±1.14	1.24±0.97	-	-
Theoretical plates	6162.167±1.58	10002.17±0.89	-	-
Repeatability of measurement (n^b^=6)	1.75	1.93	0.38	0.43

a RSD is the relative standard deviation,

b n is the number of determinations, FLX is fluoxetine HCl and OLZ is olanzapine

To optimize the HPTLC parameters, several mobile phase compositions were tried. Acetone:methanol:triethyleamine (5:3:0.5, v/v/v), gave a sharp and symmetrical peaks of FLX and OLZ with R_f_ values of 0.58±0.02 and 0.77±0.01, respectively (fig. 2). Well-defined spots (and peaks) were obtained when the chamber was saturated with mobile phase vapour for 20 min at room temperature (25°). A linear relation was obtained between peak area and the concentration of the two drugs in the range of 300-1000 ng/spot and 150-500 ng/spot for FLX and OLZ, respectively. The linear regression equations were computed as: Y= 4.4869X+182.44, r= 0.9961 and Y= 11.836X+32.073, r= 0.9964, where Y is the area under the peak, X is the concentration in μg/ml, and r is the correlation coefficient.

**Fig. 2:**
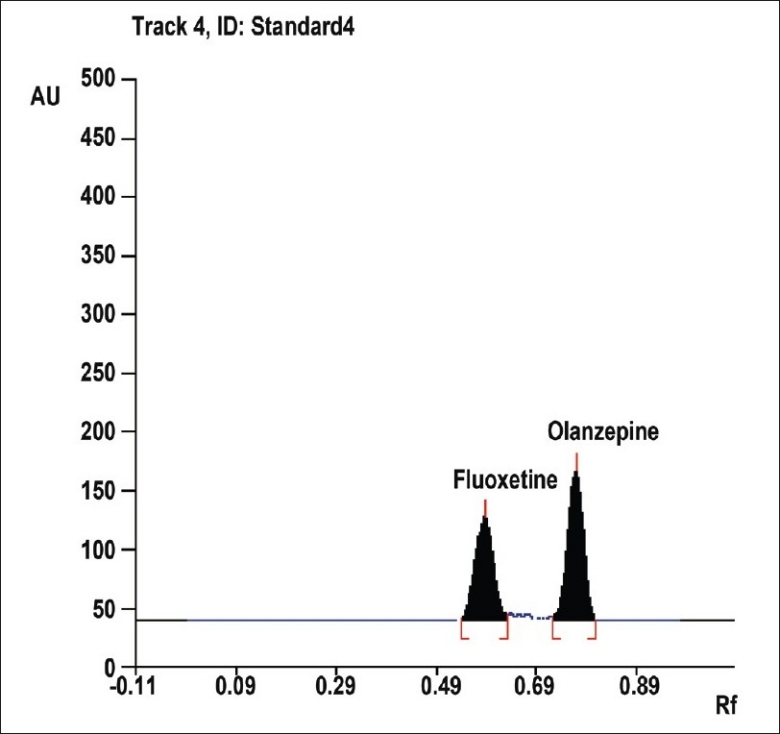
HPTLC chromatogram of FLX and OLZ at 235 nm FLX is fluoxetine HCl and OLZ is olanzapine

Results obtained by applying the RP-HPLC and HPTLC procedures showed that both the drugs can be simultaneously analyzed in the prepared mixtures with mean recoveries of 101.16±0.59 and 99.79±0.56%; 100.95±0.52 and 99.31±0.51%, respectively (Table 2). The proposed method has been applied to assay FLX and OLZ in tablets without any interference from the additives (Table 3). The results of assay validation of the proposed methods show that they are accurate and precise according to the RSD values of intra and interday determinations (Table 4).

**TABLE 2:** STANDARD ADDITION OF FLX AND OLZ TO TABLETS BY THE PROPOSED RP-HPLC AND HPTLC METHODS

Proposed methods	Concentration of drug taken (μg/ml or ng/spot)		Concentration of drug added (μg/ml or ng/spot)		Concentration of drug found (μg/ml or ng/spot)		% Recovery (n^a^=3) ± SD^b^	
				
	FLX	OLZ	FLX	OLZ	FLX	OLZ	FLX	OLZ
RP-HPLC	1	0.5	0.5	0.25	1.520	0.747	101.30±0.73	99.62±0.44
	1	0.5	1	0.5	2.019	0.997	100.94±0.59	99.72±0.72
	1	0.5	1.5	0.75	2.53	1.25	101.25±0.45	100.05±0.53
RP-HPLC	200	100	100	50	303.12	149.03	101.04±0.31	99.36±0.46
	200	100	200	100	403.71	198.45	100.93±0.84	99.23±0.55
	200	100	300	150	504.42	248.34	100.88±0.41	99.33±0.53

a n is the number of determinations,

b SD is the standard deviation, FLX is fluoxetine HCl and OLZ is olanzapine

**TABLE 3:** ASSAY RESULTS FOR TABLETS USING THE PROPOSED RP-HPLC AND HPTLC METHODS

Formulation	Proposed methods	Amount of drug taken (mg)		Amount of drug found (mg)		% Amount found (n^a^=3) ± SD^b^	
		
		FLX	OLZ	FLX	OLZ	FLX	OLZ
Tablets	RP-HPLC	20	10	20.123	9.922	100.62 ± 0.29	99.23 ± 0.31
		20	10	20.23	9.897	101.13 ± 0.62	98.98 ± 0.97
	HPTLC	20	10	20.153	9.938	100.75 ± 0.61	99.38 ± 0.60
		20	10	20.119	9.989	100.60 0.56	99.89 0.52

a n is the number of determinations,

b SD is the standard deviation, FLX is fluoxetine HCl and OLZ is olanzapine

**TABLE 4:** SUMMARY OF VALIDATION PARAMETERS FOR THE PROPOSED RP-HPLC AND HPTLC METHODS

Parameters	Proposed methods			
	
	RP-HPLC		HPTLC	
		
	FLX	OLZ	FLX	OLZ
LOD^a^ μg/ml or ng/spot	0.00526	0.01594	3.433177	4.884
LOQ^b^ μg/ml or ng/spot	0.0106	0.032123	10.40357	14.8
Interday (n^d^ = 3) (RSD^c^, %)	0.51-1.75	0.36-1.65	0.28-1.60	0.37-1.33
Intraday (n^d^ = 3) (RSD^c^, %)	0.18-1.51	0.39-1.37	0.29-1.22	0.39-1.22

a LOD is the limit of detection,

b LOQ is the limit of quantification,

c RSD is the relative standard deviation,

d n is the number of determinations, FLX is fluoxetine HCl and OLZ is olanzapine

The assay results for FLX and OLZ in their tablet dosage form obtained using the RP-HPLC and HPTLC methods were compared by applying the paired t-test. The calculated t-values 0.60 for FLX and 0.49 for OLZ are less than the tabulated t-value (3.82) at the 98% confidence interval. Therefore, there is no significant difference in a determined content of FLX and OLZ by the RP-HPLC and HPTLC methods. The proposed procedures can be applied for the simultaneous determination of FLX and OLZ. Moreover, the methods are rapid, sensitive, accurate, precise and can be used in routine analysis.

## References

[1] O'Neil MJ (2006). The Merck Index.

[2] Mishra L (2006). Drug Today.

[3] (2005). British Pharmacopoeia.

[4] (2005). The United States Pharmacopoeia.

[5] Bebawy LI, El-Kousy N, Suddik JK, Shokry M (1999). Spectrophotometric determination of fluoxetine and sertraline using chloranil, 2,3-dichloro-5,6-dicyanobenzoquinone and iodine. J Pharm Biomed Anal.

[6] Abbas A, Tayyebeh M, Lida K (2006). Spectrophotometric determination of fluoxetine by batch and flow injection methods. Chem Pharm Bull.

[7] Naik MT, Rokade MD, Dhadke PM (1999). Extractive spectrophotometric estimation of fluoxetine hydrochloride in pharmaceutical formulations. Indian J Pharm Sci.

[8] Peyton AL, Carpenter R, Rutkowski K (1991). The stereospecific determination of fluoxetine and norfluoxetine enantiomers in human plasma by high-pressure liquid chromatography (HPLC) with fluorescence detection. Indian J Pharm Sci.

[9] Raggi MA, Mandrioli R, Casamenti G, Volterra V, Desiderio C, Fanali S (1999). Improved HPLC determination of fluoxetine and norfluoxetine in human plasma. J Chromatogr.

[10] Maanni A, Combourieu I, Bonini M, Creppy EE (1993). Fluoxetine, an antidepressant, and norfluoxetine, its metabolite, determined by HPLC with a C8 column and ultraviolet detection. J Clin Chem.

[11] Green R, Houghton R, Scarth J, Gregory C (2002). Determination of fluoxetine and its major active metabolite norfluoxetine in human plasma by liquid chromatography-tandem mass spectrometry. J Chromatogr.

[12] Berzas JJ, Guiberteau C, Contento AM, Rodriguez V (2002). Sensitive and rapid high-performance liquid chromatographic method for simultaneous determination of antidepressants in pharmaceutical formulations. J Chromatogr.

[13] Juan H, Zhiling Z, Huande L (2005). Simultaneous determination of fluoxetine, citalopram, paroxetine, venlafaxine in plasma by high performance liquid chromatography–electrospray ionization mass spectrometry (HPLC-MS/ESI). J Chromatogr B.

[14] Berzas Nevado JJ, Villasen Llerena MJ, Contento Salcedo AM, Aguas Nuevo E (2000). Determination of fluoxetine, fluvoxamine, and clomipramine in pharmaceutical formulations by capillary gas chromatography. J Chromatogr Sci.

[15] (2007). Indian Pharmacopoeia.

[16] Krebs A, Starczewska B, Puzanowska-Tarasiewicz H, Sledz J (2006). Spectrophotometric determination of olanzapine by its oxidation with N-bromosuccinimide and cerium (IV) sulphate. Anal Sci.

[17] Raggi MA, Casamenti G, Mandrioli R, Izzo G, Kenndler E (2000). Determination of the novel antipsychotic drug olanzapine in human plasma using HPLC with amperometric detection. J Pharm Biomed Anal.

[18] Jasinska A, Starczewska B, Polkowska M, Puzanowska Tarasiewicz H (2005). Solid phase extraction of olanzapine with reverse phase sorbents prior to UV and HPLC analysis. Anal Lett.

[19] Raggi MA, Casamenti G, Mandrioli R, Fanali S, Ronchi DD, Volterra V (2000). Determination of the novel antipsychotic drug olanzapine in human plasma using HPLC with amperometric detection. J Chromatogr.

[20] Llorca PM, Coudore F, Corpelet C, Buyens A, Hoareau M, Eschalier A (2001). Integration of olanzapine determinations in a HPLC-diode array detection system for routine psychotropic drug monitoring. J Clin Chem.

[21] Berna M, Ackermann B, Ruterbories K, Glass S (2002). Determination of olanzapine in human blood by liquid chromatography-tandem mass spectrometry. J Chromatogr B.

[22] Shah CR, Shah NJ, Suhagia BN, Patel NM (2007). Simultaneous assay of olanzapine and fluoxetine in tablets by column high-performance liquid chromatography and high-performance thin-layer chromatography. J AOAC Int.

[23] Reddy BV, Reddy KVNS, Sreeramulu J, Kanumula GV (2007). Simultaneous determination of olanzapine and fluoxetine by HPLC. J Chromatogr.

